# Supplementary material for: Identification of photoperiod-induced specific miRNAs in the adrenal glands of Sunite sheep (Ovis aries)
Source: Front Vet Sci. 2022 Jul 22;9:888207. doi: 10.3389/fvets.2022.888207 (PMC9354845; doi:10.3389/fvets.2022.888207)
Supplement: Supplementary file 1 [file Data_Sheet_1.ZIP › Supplementary file/miRDeep2 file.zip!/miRDeep2 file/Know_Structure/SP42A1/oar-mir-26a.pdf]

miRBase precursor : oar-mir-26a
Total read count : 960340
oar-miR-26a read count : 960252
remaining reads : 88

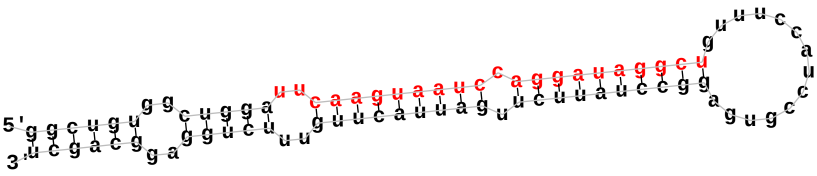

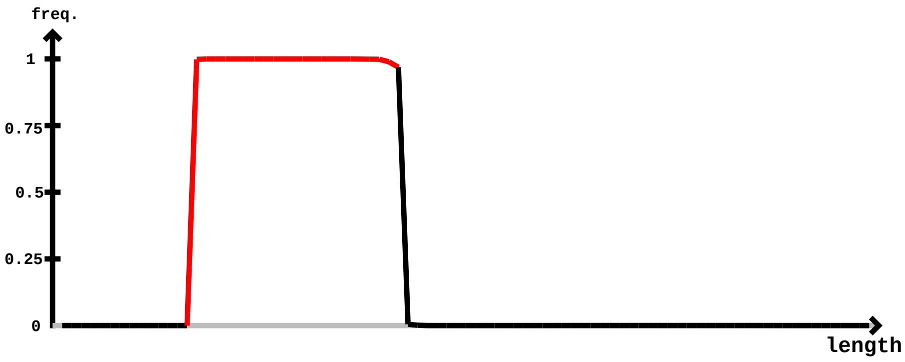

oar-miR-26a

5'- ggcuguggcuggauucaaguaauccaggauaggcuguuuccauccgugaggccuauucuugauuacuuguuucuggaggcagcu -3' exp

((((((..(((((..(((((((((.((((((((((..............))))))))))..)))))))))..)))))..))))))	reads	mm	sample
............auucaaguaauccaggauaggc..	2	0	seq
............auucaaguaauccaggauaggcu...	37	0	seq
............Guucaaguaauccaggauaggcu...	5	1	seq
.............uucaUguaauccaggau..	2	1	seq
.............uucaaguaauccaggaG..	5	1	seq
.............uucaagGaauccaggau..	1	1	seq
.............uGcaaguaauccaggau..	1	1	seq
.............uucaGguaauccaggau..	1	1	seq
.............uucaaguaauccaggaA..	9	1	seq
.............uucaaguaauccaggau..	337	0	seq
.............uAcaaguaauccaggau..	1	1	seq
.............uucaaguaauccaggaua...	484	0	seq
.............uucaGguaauccaggaua...	3	1	seq
.............uucaaguaauccaggauaC..	5	1	seq
.............uucaaguaauccaggauag..	437	0	seq
.............uucaaguaauccaggauagg...	8928	0	seq
.............uucaaguaauccaggauaggc..	19744	0	seq
.............uucaaguaauccaggauaggcu...	923743	0	seq
.............uucaaguaauccaggauaggcug..	3516	0	seq
.............uucaaguaauccaggauaggcugu...	1208	0	seq
.............uucaaguaauccaggauaggcuguu..	17	0	seq
.............uucaaguaauccaggauaggcuguG..	2	1	seq
.............uucaaguaauccaggauaggcuguuu...	6	0	seq
..............ucaaguaauccaggaua...	1	0	seq
..............ucaaguaauccaggauag..	2	0	seq
..............ucaaguaauccaggauagg...	11	0	seq
..............ucaaguaauccaggauaggc..	29	0	seq
..............ucaaguaauccaggauaggcu...	1604	0	seq
..............ucaaguaauccaggauaggcug..	13	0	seq
..............ucaaguaauccaggauaggcugu...	5	0	seq
...............caaguaauccaggauagg...	1	0	seq
...............caaguaauccaggauaggc..	3	0	seq
...............caaguaauccaggauaggcu...	66	0	seq
...............caaguaauccaggauaggcug..	1	0	seq
................aaguaauccaggauaggcu...	21	0	seq

oar-miR-26a

```
ggcuguggcuggauucaaguaauccaggauaggcuguuuccauccgugaggccuauucuugauuacuuguuucuggaggcagcu
..................guaauccaggauaggcu.................................................    1     0     seq
................................................aggccuauucuugauuacuuguu.............    3     0     seq
................................................aggccuauucuugauuacuuguuu............    3     0     seq
...................................................ccuauucuugauuacuuguu.............    4     0     seq
...................................................ccuauucuugauuacuuguuu............    29    0     seq
...................................................ccuauucuugauuacuuguuuc...........    45    0     seq
...................................................ccuauucuugauuacuuguuucu..........    3     0     seq
....................................................cuauucuugauuacuuguuuc...........    1     0     seq
```
